# *Mycoplasma gallisepticum* and *Mycoplasma synoviae* in Turkeys in Poland

**DOI:** 10.3390/pathogens13010078

**Published:** 2024-01-15

**Authors:** Olimpia Kursa, Grzegorz Tomczyk, Agata Sieczkowska, Sylwia Kostka, Anna Sawicka-Durkalec

**Affiliations:** Department of Poultry Diseases, National Veterinary Research Institute, 24-100 Puławy, Poland; gtomczyk@piwet.pulawy.pl (G.T.); agata.sieczkowska@piwet.pulawy.pl (A.S.); sylwia.kostka@piwet.pulawy.pl (S.K.); anna.sawicka@piwet.pulawy.pl (A.S.-D.)

**Keywords:** *Mycoplasma synoviae*, *Mycoplasma gallisepticum*, turkeys, respiratory tract

## Abstract

The pathogenic mycoplasmas are among the bacteria causing significant losses in the poultry industry worldwide. *Mycoplasma gallisepticum* (MG) and *M. synoviae* (MS) are economically important pathogens causing chronic respiratory disease, decreased growth, egg production and hatchability rates, and significant downgrading of carcasses. Effective diagnosis of infection with these species in poultry is highly requisite considering their two routes of spreading—horizontal and vertical. Their prevalence and molecular epidemiology were investigated in 184 turkey flocks in Poland. Tracheal samples were selected from 144 broiler flocks and 40 turkey breeder flocks collected in 2015–2023. The prevalence of MG was determined by real-time PCR targeting the 16S rRNA gene and PCR targeting the *mgc2* gene, and MS was determined by a 16–23S rRNA real-time PCR and a *vlh*A gene PCR. Further identification and molecular characterization were carried out using PCR and sequencing. *M. gallisepticum* and *M. synoviae* were found in 8.33% and 9.72% of turkey broiler flocks respectively. The phylogenetic analysis of MG isolates in most cases showed high similarity to the ts-11-like strains. MS isolates showed high similarity to strains isolated from flocks of laying hens causing EAA. Additional tests detected *Ornithobacterium rhinotracheale*, *Gallibacterium anatis*, *Enterococcus faecalis* and *Enterococcus faecium, Staphylococcus aureus* and *Riemerella anatipestifer*. These secondary pathogens could have significantly heightened the pathogenicity of the mycoplasma infections studied.

## 1. Introduction

*Mycoplasma synoviae (MS)* and *M. gallisepticum (MG)* belong to the Mollicutes class and *Mycoplasmataceae* family. They are bacteria with pleomorphic cells lacking walls and have highly variable surface proteins. They are the smallest self-replicating prokaryotes [1,2]. The pathogenicity of *M. synoviae* and *M. gallisepticum* depends on many factors related to the bacterial strain and the age, stress exposure and immune status of the bird [3,4]. Co-infection with other bacteria or viruses that damage the respiratory tract can worsen the severity of disease symptoms and suppress immunity. Mixed infection with viruses causing infectious bursal disease or avian influenza and bacteria such as *E. coli, Ornithobacterium rhinotracheale, Avibacterium paragallinarum* and *Gallibacterium anatis* may increase the rate of disease, resulting in increased morbidity and mortality in flocks [5,6,7,8,9]. Typically, mixed infections are responsible for *M. gallisepticum* and *M. synoviae* causing outbreaks of chronic respiratory disease (CRD) in chickens and infectious sinusitis in turkeys [9,10]. In addition, as with many other pathogens, environmental factors can also exacerbate symptoms of these diseases. The clinical form of the disease causes economic losses as a consequence of respiratory problems and reproduction disorders in birds. *M. gallisepticum* is responsible for causing nasal discharge and foamy eye secretions, often preceded by swelling of the infraorbital sinuses, which may result in partial or complete eye closure. Additionally, tracheal rales, coughing, dyspnea, listlessness, decreased feed intake and weight loss occur [1,9,10,11,12]. *M. synoviae* can cause joint lesions and respiratory signs, but also a wide range of pathological changes in the ovaries and oviducts of laying chickens. Infection of the reproductive system of chickens with MS can reduce egg production by 10–23%, and can be responsible for changes diminishing eggshell quality by causing eggshell apex abnormalities (EAAs), which are signified by translucency at the tip of the eggs [2,13,14,15].

*M. gallisepticum* and *M. synoviae* are widespread worldwide and have been isolated from birds apart from chickens and turkeys, having been detected in other poultry species farmed on a large scale such as geese and ducks, and also in ostriches, partridges, quail, pheasants, guinea fowl and wild birds [16,17,18].

They have been isolated in many countries in Europe, Asia and Africa, as well as in the United States [2]. One of the tasks of the poultry farmer is to keep flocks healthy and free of diseases, including those caused by mycoplasmas. Unfortunately, this is very difficult because of the easy transmission of these pathogens. The widespread prevalence of MS and MG in poultry appears to be related to the route of its transmission in flocks. Mycoplasmas can spread horizontally and vertically in a flock. They can be transmitted by flock birds, personnel working on the farm or wild birds [10,19,20,21]. The often subclinical nature of mycoplasma infection does not help in its diagnosis. Environmental factors exacerbated by deficient maintenance of the birds, or factors in the birds themselves, such as age, can trigger the development of the associated diseases [19,20,21,22]. Even if birds have recovered from a clinical form of mycoplasma infection and have some degree of immunity, they can still carry the bacteria and transmit it both horizontally and vertically. Unfortunately, infected birds remain carriers for the rest of their lives [14]. Carriers must be eliminated, and this in combination with the reduced feed intake, lower egg production efficiency and higher medication costs make infections with MS and MG some of the costliest disease problems in poultry production.

Diagnostics related to the detection of *M. gallisepticum* and *M. synoviae* infections can be based on serological methods and on culture methods. In the serological method, there may be cross-reactions or nonspecific reactions, while the second method is time-consuming and results may be difficult to obtain through the presence of environmental contaminants in the sample [14]. Molecular methods are a universal tool for determining the presence of mycoplasma infections. A wide range of molecular methods based on detection, genotyping and differentiation of field and vaccine strains enables precise analysis of obtained mycoplasma isolates [23,24,25,26,27].

Knowledge of the multi-infections associated with *M. synoviae* and *M. gallisepticum* in poultry flocks is constantly being supplemented. Studies conducted around the world have revealed a large number of factors involved in colonization, development of infection and manifestation of clinical signs caused by the entry of mycoplasmas into the bird’s body. The objective of this study was to initially determine the prevalence of *M. synoviae* and *M. gallisepticum* in flocks of turkeys using randomly selected samples from material collected over a period of several years. This article also focusses on co-infections with the most common bacterial respiratory pathogens.

## 2. Materials and Methods

### 2.1. Sample Collection

A total of 300 flocks from different parts of Poland were examined for *M. synoviae* and *M. gallisepticum* infection. Tracheal swab samples were brought to the Department of Poultry Diseases at the National Veterinary Research Institute in Poland as part of a routine diagnostic test and monitoring programme. Samples from 184 turkey flocks, 144 broilers (10 swab samples per flock) and 40 breeders (60 swab samples per flock) were randomly selected from samples collected in 2015–2023. All examined samples came from birds which had been floor reared, and most of them had shown no clinical respiratory or reproductive signs. Some of the broilers had manifested respiratory signs in the form of rales and coughing, and some of them had displayed neurological signs and consumed less feed. The turkey flocks were not vaccinated.

### 2.2. DNA Extraction

Tracheal swabs from each flock were pooled separately into tubes containing Tris-EDTA buffer and processed for DNA extraction. Genomic DNA was extracted using a QIAamp DNA Mini kit (Qiagen, Hilden, Germany) according to the manufacturer’s recommendations. The quantity and quality of the DNA was determined using the NanoDrop 1000 system (Thermo Scientific, Waltham, MA, USA). The solution obtained by using the DNA extraction procedure on the Tris-EDTA used for sample preparation was conducted as a negative control. Samples were frozen at −20 °C until future analysis.

### 2.3. Real-Time PCR

The presence of DNA of *M. synoviae* was detected by real-time PCR with primers complementary to the 16S-23S intergenic spacer region, and the presence of DNA of *M. gallisepticum* was also detected by real-time PCR with primers complementary to the highly conserved *mgc2* gene as described previously by Raviv et al., with slight modifications [28]. The reaction was carried out using a QuantiTect Probe PCR Kit (Qiagen) with 12.5 μL of master mix, 1.3 μL of each 10 μM primer, 0.5 μL of probe, 7.4 μL of distilled water and 2 μL of DNA in a total volume of 25 μL in an ABI 7500 thermal cycler (Applied Biosystems, part of Thermo Fisher Scientific, Waltham, MA, USA) initially at 95 °C for 3 min, and then through 40 cycles at 95 °C for 3 s. The fluorescence data were collected during a 60 °C, 32 s annealing–extension step.

### 2.4. PCR and Sequence Analysis

The PCR for *M. synoviae* was conducted using previously described specific primers which amplify the *vlh*A gene [24]. For *M. gallisepticum,* the *mgc2* gene primers used were described by Ferguson et al. [25]. The PCR assays were performed on positive samples obtained in the real-time PCR. The reaction mixture contained Taq PCR Master Mix (EURx, Gdańsk, Poland) in a volume of 12.5 μL, 1.5 μL of each 10 μM primer, 7.5 μL of distilled water and 2 μL of DNA to give a total reaction volume of 25 μL. The PCR procedure included an initial incubation at 95 °C for 1 min, 35 cycles at 95 °C for 40 s each, annealing at 50 °C for 40 s, extension at 72 °C for 40 s and a final extension at 72 °C for 2 min. DNA sequence analysis of three gene fragments was also performed. The putative phase-variable adhesin protein (*pvpA*) gene, the intergenic 16S- 23S rRNA (IGSR) gene and the *M. gallisepticum* cytadhesin 2 (*mgc2*) gene used the primers and procedures described by Ferguson et al. [29]. The PCR amplicons were separated by electrophoresis on a 2% agarose E-gel plate containing ethidium bromide (Invitrogen, part of Thermo Fisher Scientific) and were visualised by ultraviolet transillumination (Invitrogen). The identification of MS and MG was confirmed by sequencing the amplified fragments. Selected PCR products were sent for sequencing by the Sanger method to a commercial service (Genomed, Warsaw, Poland). Closely related sequences of MS and MG were downloaded from GenBank. Multiple sequence alignments were established, and phylogenetic trees were constructed using Clustal W in MEGA 11 software and the neighbour-joining tree-inference method, with evolutionary distances computed using the maximum-likelihood method with 1000 bootstrap replicates [26]. 

### 2.5. Presence of Other Bacterial Pathogens

Turkey flocks positive for MG and MS from field outbreaks were tested for *Ornithobacterium rhinotracheale* via real-time PCR targeting the 16S rRNA gene according to Abdelwhab et al. [27]. Positive turkey flocks were tested for *M. meleagridis* also in a PCR targeting 16S rRNA [18]. Positive flocks were also tested for *Gallibacterium anatis* once again using a PCR targeting 16S rRNA [28]. A 10 μL volume of supernatant of the swabs from samples positive through PCR was inoculated onto a MacConkey agar, a tryptic soy agar, a bile esculin azide agar and a Columbia agar plate with 5% sheep’s blood. The plate with MacConkey agar was incubated at 37 °C for 24 h. The plates with tryptic soy agar, bile esculin azide agar and Columbia agar were incubated at 37 °C under a 5% CO_2_ atmosphere for 24 h. The obtained colonies were verified by matrix-assisted laser desorption/ionisation–time-of-flight mass spectrometry (MALDI-TOF MS). The bacterial colonies from the agar plates were transferred to the MALDI target plate and mixed with formic acid and an α-cyano-4-hydroxycinnamic acid matrix solution. All mass spectra were analysed with Bruker Daltonics software v.4.2 (Bruker Corporation, Billerica, MA, USA).

### 2.6. Statistical Analysis

Spearman’s rank correlation coefficient test was used to compare the results obtained using the two methods. The value of *p* < 0.05 was considered statistically significant. Statistical analyses were performed using the Social Science Statistics program (www.socscistatistics.com 15 November 2023).

## 3. Results

### 3.1. Isolation and Identification

All the MS and MG real-time PCR and PCR results from the study are summarised in Table 1. All samples positive in the real-time PCR were subjected to a specific PCR, in which not all of them gave amplifications. However, most of the results obtained by the real-time method were confirmed by PCR. No significant differences were found (*p* > 0.05). MG and MS were not found in samples sent from flocks of breeding turkeys. The presence of MG in broiler flocks was found in 2015, 2016, 2019 and 2023. The average prevalence of MG in this flock of broilers was 8.33% and varied significantly from year to year. In 2015 it was 1.64%, while in 2019 it was as high as 50%. The presence of MS in broiler flocks was found in 2015, 2016 and 2019. In turkey flocks, MS DNA was found on average in 9.7%, varying from a 19.67% high in 2015 to a 6.25% low in 2019. 

### 3.2. Molecular Characterisation and Phylogenetic Relationship

Successful amplification of the region of the *vlhA* gene was achieved for MS and the *mgc2* region for MG from turkey flocks. Analysis of the results obtained from the targeted MG genes showed that the turkey broiler flocks were infected probably with a field strain. Among the 14 samples positive by real-time PCR for MS, amplicons were obtained for 13. Of those 13 sequenced MS isolates, most were isolated from turkeys without clinical signs, and 1 strain originated from flocks where clinical signs were present.

**Figure 1:**
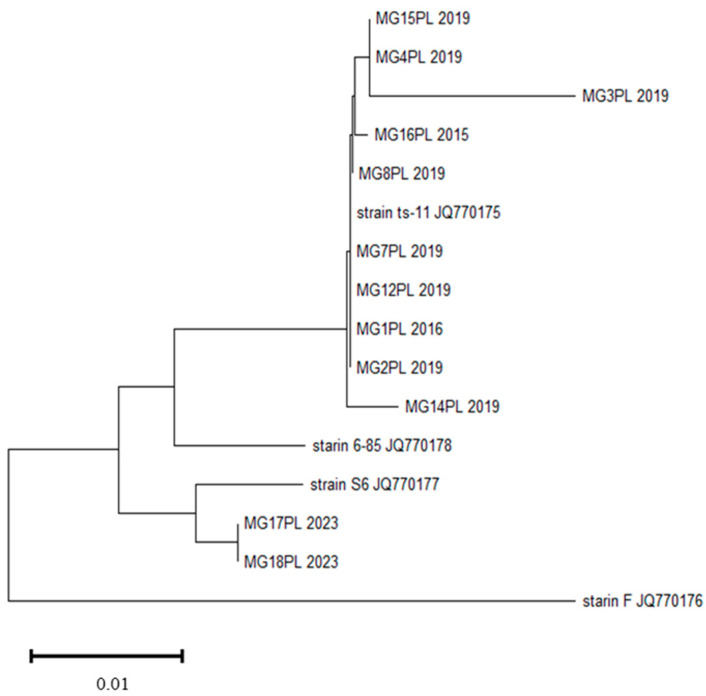
Phylogenetic tree constructed based on *mgc2* gene of MG strains isolated from turkey flocks and reference strains.

Of the 12 MG isolates obtained from flocks of broiler turkeys, most were closely related to the ts-11 vaccine strain. The dendrogram formulated from the mgc 2 sequence shows that MG strains formed two clusters. Ten MG strains formed subclusters showing high similarity to strain ts-11. These are strains isolated in 2015, 2016 and 2019. Two strains showing high similarity to each other that were isolated in 2023 formed a separate cluster (Figure 1).

**Figure 2:**
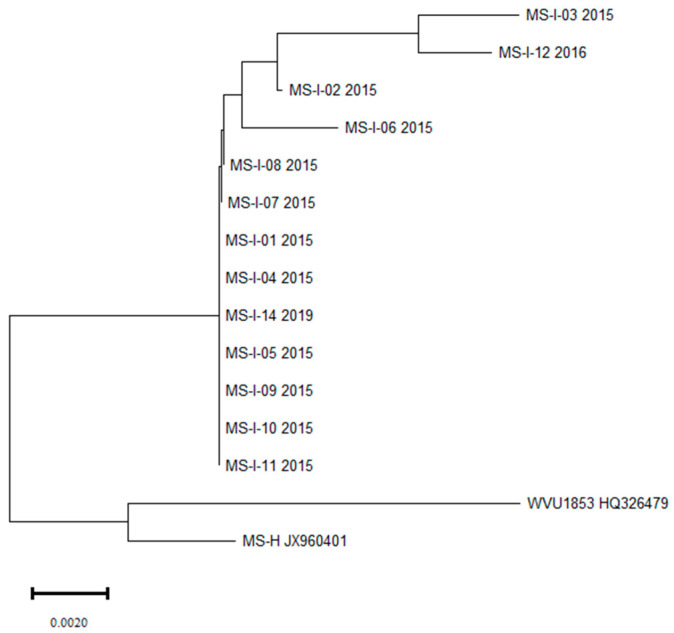
Phylogenetic tree created based on the *vlhA* gene sequences of MS strains isolated from turkey flocks, the reference strain and the vaccine strain.

Thirteen *vlhA* gene sequences formed a single clade, with four subclades completely distinct from the reference strain WVU1853 and the vaccine strain MS-H (Figure 2). Analysis of the *vlhA* gene sequences showed that Polish MS strains from turkeys show high similarity to the sequences from many MS cases detected in recent years, mainly in poultry in Poland and other countries.

### 3.3. Multi-Infection with Bacterial Pathogens

The presence of the DNA of other respiratory tract bacteria was found in some of the tested trachea swab samples. Diagnostic tests by molecular methods showed the presence of *Ornithobacterium rhinotracheale* (ORT) (88.46%) and *Gallibacterium anatis* (GA) (30.77%) in samples collected from turkeys. The results are presented in Table 2. Bacterial colony growth was obtained in all samples that were positive for MS and MG, and those colonies were identified by MALDI-TOF MS. Some samples showed contamination related to bacterial overgrowth by *Proteus* sp. Tracheal swabs of turkeys showed the presence of pathogenic bacteria that can exacerbate disease symptoms, including the haemolytic biovar *Gallibacterium anatis*, *Enterococcus faecalis* (23.08%), *Enterococcus faecium* (7.69%), *Streptococcus aureus* (3.85%), *Riemerella anatipestifer* (3.85%) and *E. coli.* These secondary pathogens may play a significant role in the pathogenicity of current mycoplasma infections. The occurrence of *M. meleagridis* in turkeys can cause major losses, especially in mixed infections with MG and MS. However, the presence of *M. meleagridis* was not detected in samples from turkeys. 

## 4. Discussion

*M. gallisepticum* and *M. synoviae* are known worldwide to be virulent pathogens, and they are listed and notifiable to the World Organisation for Animal Health (OIE) [30]. Rules have been established by EU Regulation (EU) 2016/429 (Animal Health Law) and Regulation 2019/2035 for the monitoring of poultry and hatching eggs and their intracommunity trade and importation from third world countries, and these rules are intended to prevent the spread of MG infections. Many European countries have implemented prevention and control programmes based on strict biosecurity, diagnostic surveillance and elimination of infected flocks [31,32]. However, there are disease vectors and factors that favour the rapid recurrence of MG and MS outbreaks in the rapidly developing turkey sectors of the poultry industry. Given that Poland is one of the largest poultry producers in Europe, keeping flocks free of infection is a big challenge. One way to prevent mycoplasma infections is vaccination. In the Polish poultry industry, the most commonly used vaccines are preparations containing live cultures of 6/85 [33] or ts-11 [34] MG strains and of the MS-H strain [35]. Vaccination of flocks increases their resistance to infection with wild-type strains. However, ongoing studies have shown that bacterial vaccination only reduces, and does not eliminate, colonisation by MG after a challenge [2]. In our study, we found ts-11-like isolates in flocks of turkey broilers. In all cases, the birds were not vaccinated and came from different locations. The broilers may have been exposed to the vaccine accidentally through contact with staff or equipment. The flocks probably had horizontal infection. A similar case has been described previously in broiler hens [36]. Ongoing studies with the ts-11 vaccine indicate that the vaccine has minimal or no virulence for hens and turkeys, and induces good protection against MG infections. 

Among the many factors affecting the development of infection with pathogenic mycoplasmas are host-related factors such as age, stress or hormones. In flocks of laying hens, one factor is the timing of the birds’ entry into the laying period, which is a considerable strain on the bird’s body, and also the period when birds are most susceptible to infection or the manifestation of disease symptoms [19,21]. Environmental factors such as seasonal changes or cold stress are also major potential factors in the development of the clinical form of mycoplasma-associated diseases [37,38,39]. The disease vector involving contact with wild birds is a factor that allows transmission of pathogens to other birds. The epidemiological status of *M. synoviae* infection depends largely on the pathogenicity of the strain, the route of infection and its predilection for host organs. Strains of MS can cause respiratory and locomotor symptoms (the latter causing arthritis) and affect the reproductive system, leading to EAA in hens [13,40,41,42]. In this study, MS was isolated mostly (*n* = 12) from flocks where there were no severe clinical symptoms other than decreases in feed intake. As for the pathogenicity of MG strains, in most cases (*n* = 8) it is seen in respiratory symptoms of differing severity co-occurring with neurological problems. This may be due to the presence of multi-infection in the flock because ORT was also found in these samples. The presence of mixed infection with a pathogenic strain of ORT may have additionally increased the symptoms of the disease. Clinical signs occurring in MG and MS infections may be related to the presence of secondary bacterial factors such as mixed infection with *Ornithobacterium rhinotracheale, E. faecalis, R. anatipestifer* or *E. coli,* which were also detected in the flocks. The impact of co-infection on the course of mycoplasma infection is reported in many publications [6,36,43,44]. These include multi-infections with bacterial agents such as *Avibacterium paragallinarum* and *Gallibacterium anatis* besides MG and MS. Viral agents may also facilitate the development of the clinical form of the diseases associated with mycoplasma infection [45]. Pathogenic bacteria subclinically infecting the respiratory system of birds may not be detected in the absence of factors influencing the development of the disease. However, clinical symptoms caused by MG and MS infections, as well as multi-infections, in most cases produce similar symptoms of the disease, which may affect proper diagnosis and treatment.

In this study, we focused on the molecular characterisation of MG and MS isolated from poultry flocks in Poland. Molecular methods are widely used, sensitive, specific and fast techniques regarded as alternatives to culture and serological methods in poultry diagnostics. This study presents, for the first time, preliminary data acquired using these techniques on isolates of MG and the current situation related to the prevalence of infections of MS in Polish turkey flocks. Genetic material of MG and MS was found in flocks of broiler turkeys. The prevalence of MG and MS was found to be quite low. The incidence of MG in turkey flocks was 8.33%. Of these, 83.3% (*n* = 10) were ts-11-like isolates. Comparing the phylogenetic tree obtained from MG cases, these strains showed the greatest homology to strains circulating in poultry in Europe, Asia, the USA and China. Two isolates showed 97.94% similarity to a strain isolated from hens in Saudi Arabia in 2016 (MG149559.1), or to an isolate from Pakistan from hens in 2017 (KY126378.1). The incidence of MS in turkey flocks was higher than that of MG and was 9.72%. The genetic analysis of Polish MS field isolates collected from turkeys during the period 2015–2023 was based on the partial *vlhA* gene sequence. The field strains showed the greatest similarity to strains isolated at the same time in Poland in 2015 and 2016 from flocks of laying hens showing changes in shell structure (EAA). However, the tested flocks had mostly shown no clinical signs, which, if such inconspicuousness is typical, is unfavourable to poultry production, as the infection remains silent and can spread unnoticed. Relatedness of the MS strains with nucleotide sequence homology ranging from 99.26 was noted with KM985992 isolated from hens in South Korea and KC832823 isolated from flocks of turkey broilers in Italy. The low level of MG and MS infections in turkey flocks in Poland may be related to various aspects. National monitoring programs, widespread vaccinations as well as the level of biosecurity have a significant impact on keeping poultry flocks free of *M. gallisepticum* and *M. synoviae* infections.

The presence of mycoplasmas in poultry flocks has been reported in various European countries such as Germany, Italy, UK and Belgium, but also outside Europe in Turkey, Morocco, Egypt and the USA [2,31]. The prevalence of MG found in turkey flocks in this study is lower than that reported in other countries, for example Egypt, where MG prevalence was found to be 70% in broilers, 40% in layers and 83% in turkeys; China, where it was 75% in commercial broiler hens; Italy (specifically Sicily), where prevalence in commercial hens was 28.6%; Turkey (28.8%) in turkeys; or Pakistan (36.8% in broilers and 29.5% in layers) [46,47,48,49,50]. In contrast, while the incidence of MS obtained in turkey flocks in this study was lower than that in commercial broiler hens in China, where the rate was 45.6%, in Sicily in commercial hens where 42.8% positivity was noted, it was higher than in Belgium where in broiler flocks 12.9% were MS positive [17,47,48]. The level of poultry infections varies in individual countries. The differences in the occurrence of MG and MS infections in different countries may be caused by the way birds are kept or the level of biosecurity. Maintaining high security standards is crucial to keep herds free of mycoplasma infections. 

## 5. Conclusions

In this study, we have shown the prevalence of *M. gallisepticum* and *M. synoviae* in breeder and broiler turkeys flocks in Poland. Considering that the majority of MS- and MG-positive samples were detected in broiler flocks, prophylaxis of breeder flocks would seem to be effective in these flocks. However, the low prevalence rate in flocks of broilers also indicates good levels of biosecurity. In birds in which wild strains of MS and MG were found, there were also other respiratory pathogens that could have exacerbated clinical signs. The low prevalence of field strains in turkeys in Poland compared to other countries allows us to conclude that the level of biosecurity in the national poultry industry is high.

## Figures and Tables

**Table 1:** Presence of MG and MS in broiler turkey flocks.

Year	Number of Samples from Broilers	MG-Positive Samples		MS-Positive Samples	
		%	*n*	%	*n*
2015	61	1.64%	1	19.67%	12
2016	15	6.67%	1	6.67%	1
2017	10	-	-	-	-
2018	6	-	-	-	-
2019	16	50%	8	6.25%	1
2020	5		-	-	-
2021	11		-	-	-
2022	0		-	-	-
2023	20	10%	2	-	-
Total	144	8.33%	12	9.72%	14

**Table 2:** Pathogens presents in flocks positive for MG and MS.

Year	MS	MG	ORT	GA	*E. faecalis*	*E. faecium*	*S. aureus*	*R. anatipestifer*
2015	12	1	10	2	2	0	0	0
2016	1	1	2	1	2	0	0	0
2019	1	8	9	4	0	0	0	0
2023	0	2	2	1	2	2	1	1
Total	14	12	23	8	6	2	1	1
Total%	9.72	8.33	88.46	30.77	23.08	7.69	3.85	3.85

## Data Availability

The data presented in this study are available on request from the corresponding author. The data are not publicly available because of legislation protecting privacy.
